# Optimal sizing of residential photovoltaic and battery system connected to the power grid based on the cost of energy and peak load

**DOI:** 10.1016/j.heliyon.2023.e14414

**Published:** 2023-03-09

**Authors:** Mohammad Vahabi Khah, Rahim Zahedi, Reza Eskandarpanah, Amir Mohammad Mirzaei, Omid Noudeh Farahani, Iman Malek, Nima Rezaei

**Affiliations:** a Department of Renewable Energy and Environmental Engineering, University of Tehran, Tehran, Iran; b Department of Energy Systems Engineering, Islamic Azad University Science and Research Branch, Tehran, Iran; c Faculty of Materials Engineering, Tarbiat Modares University, Tehran, Iran; d Faculty of Computer Engineering and Information Technology, Faran Mehr Danesh University, Tehran, Iran

**Keywords:** Sizing, Solar energy, Renewable energy, PV battery System

## Abstract

The use of renewable energy is necessary to achieve the goals of sustainable development, and sooner or later all countries are forced to plan and make policies for the use of this equipment. Considering the growing trend of smart systems and the ability of these systems to control and use renewable resources, it is necessary to investigate how to control and optimally use these resources in smart systems. Considering the geographical conditions and significant solar energy radiation in Iran, the most suitable option for using renewable energy in residential buildings is solar energy. Among the types of solar energy used around the world, photovoltaic panels are used more due to their wide range, being cheaper than other sources of electric power from solar energy and more durable than other sources. In order to reduce widespread losses and reduce the cost of transmission and distribution, increase efficiency, the possibility of the presence of private sector investors and increase the security and stability of the power grid, distributed production of electrical energy at consumption locations using small-scale units is the most cost-effective way to use home solar panels. Also, the production of energy from wind turbines can be done in the areas where anemometer data determine it to be suitable. The combination of solar energy and wind energy can effectively reduce the need for batteries, but studies show that this combination is only economically viable when it is used on a large scale and with high powers, which requires a lot of investment. Large initial capital is one of the biggest problems of distributed production systems, so the use of artificial intelligence methods for accurate capacity determination of renewable energy production systems becomes doubly important. The economic results show that the least cost of electricity and net price cost are 0.44 $ per kWh and 15.0 million $ respectively, when the converter size was gradually changed, with a renewable fraction of 46.7%.

## 1 Introduction

The usage of fossil fuels like oil and gas has caused irreversible damage to humanity, as seen by rising greenhouse gas levels and climate change. Renewable energy consumption is intended to reduce environmental pollution, including air pollution reduction and public health improvement, in order to prevent the rise of damaging impacts. Photovoltaic (PV) technology is one of the renewable energy power generation options, and it is a significant technique for reducing energy shortages and pollution [1]. A PV system is made up of solar cells, a grid panel, and a mechanical mechanism that keeps the panel pointing in the right direction. In addition to the necessary components, battery banks with PV systems are utilized to reduce energy consumption when demand is less than energy production. Standalone PV systems and grid-tied PV systems are the two basic types of PV systems. The standalone system is appropriate when delivering energy to the consumer is sufficient. The grid-connected PV system, on the other hand, uses the grid in the absence of PV system energy. Grid-connected PV systems are now widely used all over the world. Fuzzy logic controllers (FLCs) are increasingly being used in systems with nonlinearity and uncertainty, but fine-tuning input scaling factors for FLCs is difficult, and they have a direct impact on performance measurements and controller parts [2].

Optimizing and paying attention to energy management was noticed since the 70s along with the oil crisis. The progress of the West was insignificant at the beginning, so that from 1975 to 1980, they only reduced one percent of energy consumption. From 1980 to 1985, this reduction reached 15 to 20%. In the first years, the major problems were investigated and the necessary laws were established. Tax laws can be mentioned among these laws [3]. Currently, the West has reached a point where it requires very high costs to save energy, that is, the technology used by advanced countries currently has the maximum efficiency, hence the attention to renewable energy and energy storage devices in recent years to reduce costs. Is taken into consideration [4].

In recent years, it has become increasingly common for energy research applications to use fuzzy logic controllers. Power converters are essential components of flexible modern power networks because they allow customers to transform energy into the required form. However, in most power electronic converters, full-wave rectifiers, which are inherently non-linear, are used. On the other hand, recent research indicates that non-linear loads lead to low power factor performances and may adversely affect other power grid components. Power factors can be improved with LC filters; however, they have high capacitor values and generate non-sinusoidal source currents with high harmonic content interruption and non-linear behavior. An evaluation of the important types of DC-DC boost converters is made concerning efficiency, component number, and stability. An improved power factor and ripple-free scope are the benefits of boost converters compared to other regulators, such as buck, boost, or buck-boost converters. Power grids typically use boost converters to convert energy into desired forms since they operate in continuous conduction mode (CCM) and have minimal electromagnetic interference (EMI)

A serious development of new energies began in the 80s and early 90s, during this time period, there were policies for the development of renewable energies in only a few countries, but in the period from 1998 to 2005, and especially from 2005 to 2010, the countries took a lot of action in developing strategies and policies for the development of renewable energies [5]. Today, the importance of using renewable energy is not hidden from anyone. Specifically, to deal with the phenomenon of climate change, many governments have set high goals for the development of the use of renewable energy for power generation. For example, the European Union's requirement is that 20% of all energy consumed in the Union in 2020 comes from renewable sources [6].

Smart systems will play an effective role in future energy management systems. The first experimental intelligent system was released in 1966. Common use of home automation includes lighting control, heating and cooling, security, energy consumption optimization, etc. In 2016, it has become common to see home automation systems in commercial buildings, universities or hotels, but the use of these systems in private homes is limited to the affluent section of society [7] Although currently the number of smart homes is small, in less than a decade, most of the new houses and apartments will be built with smart home technology at a very low cost for consumers. Millions of dollars were spent to build early smart homes because mostly hand-made components were used. It is predicted that during the next four years, the smart home systems market will achieve an annual growth rate of 60% between 2020 and 2024. It is also predicted that the number of home appliances that work with the smart building system will grow to more than 40 million in 2024 [8].

Considering the growth trend of renewable energies and smart systems in the world, as well as Iran's policies in order to use as much renewable energy sources and reduce energy consumption as possible, the adoption of these resources and smart systems will increase in the coming years. Nowadays, buildings in the smart grid are no longer considered as fixed and uncontrollable loads, but they are used for energy management as controllable loads. How to control these loads, choosing the optimal combination of system components, including energy sources and consumers, predicting system energy consumption and production, and integrated system management are some of the challenges facing smart homes. The use of renewable resources and energy storage and optimal planning in smart buildings can lead to a reduction in energy consumption, a reduction in operating costs, and a reduction in the production of greenhouse gases.

**Fig. 1:**
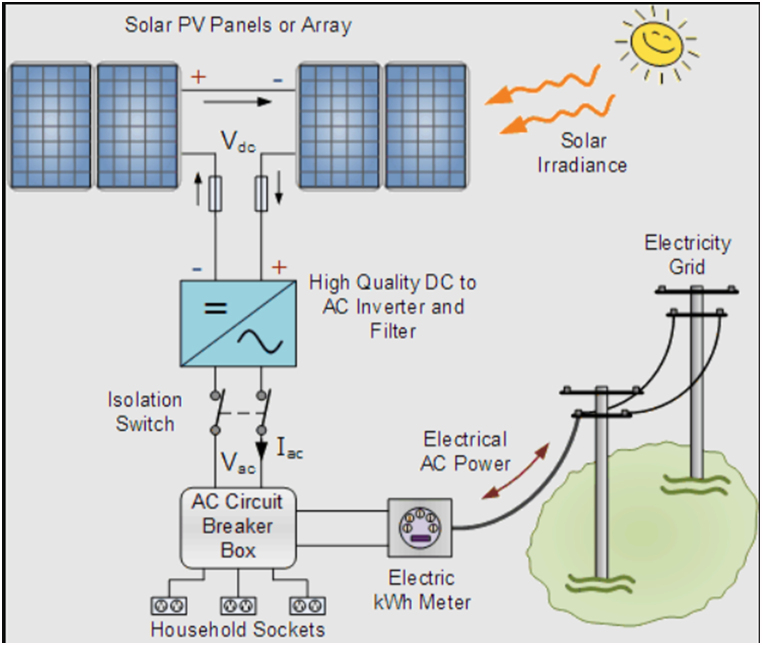
Residential section connected to the national grid and equipped with batteries and photovoltaic panels.

By taking into account the above literature, in this paper, an optimal DG placement in the distribution network is presented. In the proposed framework, two kinds of resources including fossil fuel and PV are supposed. To deal with the uncertainty of renewable resources, a point estimate method (PEM) is suggested and the planner is looking for the best location and capacity of such DGs as shown schematically in Fig. 1. The reason of PV/Battery system being the backup energy supply is its economic justification and social acceptance for Iran. Unfortunately, the fossil fuels are very cheap compared to renewable energies so renewables like solar doesn't have economic justifications for the government yet.

## 2 Types of sizing methods

### 2.1 Thumb method

This method, which is the oldest sizing method, as its name suggests, gives the desired size approximately [9]. Due to their simple, reliable design, active boost converters have been widely used to improve power factors. Parallel-connected boost converters, on the other hand, use two boost converters that are connected in parallel. By acting as an electrical switch, the converter converts an unregulated DC input voltage into a regulated output voltage. This circuit diagram shows a Boost-converter circuit using electronic circuits that waste little energy when connected to two circuits that vary the input voltage [10]. Fuzzy logic systems effectively manage non-linearity and uncertainty in designs in addition to traditional control approaches. These appropriate controllers are still excellent because they consistently respond to the system as shown in equation (1) [11].

(1) $$P_{pv}=\frac{E_{L}}{\mu_{s}.\mu_{inv}.PSH}S_{f}$$

Mamdani fuzzy models use the triangular membership function to relate each variable, for example, errors and the change in error or delta errors, to provide output. In the fuzzy logic approach, the error (e) and variations of error (e) values will be considered inputs to the fuzzy controller. The duty cycle (δ) will be output as the internal feedback control reference value.

### 2.2 Numerical method

In this method, which is more advanced and accurate than the fingertip method, PV and battery calculation is done by iterative solution [12]. By calculating the technical parameters and taking into account the minimum and background of the desired size for PV and battery, this method gives the first and lowest acceptable power for PV and battery [13].

An a FOBSMC controller based on ACO algorithm is proposed for controlling a solar system. A fractional backstepping sliding mode controller is proposed here by presenting a novel fractional order sliding surface. In the following section, the ant colony optimization method is used to optimize all controllers’ parameters.

### 2.3 Analytical method

The general form of integer calculations is fractional calculus. For fractional calculus, different definitions are given such as Riemann-Lowville (RL), Grunwald-Letnikov (GL), and Caputo [14]. In order to extract as much power as feasible from the PV module, a converter is employed to adjust the PV module output voltage V_pv_.

In this method, a graph of the decision variables is drawn in terms of all values and the best PV and battery size is selected from these graphs [15]. In this method, unlike the numerical methods, the size of PV and battery is obtained simultaneously, and the smallest amount of the desired size is selected to meet the needs of the consumer [16]. The most used software in the sector of sizing energy systems is HOMER software [17].

The correct tuning of the controller coefficients is one of the most difficult aspects of controller design. The fine-tuned controllers work more efficiently. Swarm intelligence is a problem-solving strategy based on the social behaviors of insects and other animals that is still relatively new. Ants, in particular, have sparked the development of a number of methodologies and procedures, the most well-known and effective of which being ant colony optimization, a general-purpose optimization tool. The foraging behavior of some ant species inspired ACO. Pheromones are left on the ground by these ants to indicate a preferred path for other colony members to follow. Since the first ant colony optimization. Method was proposed in early 1990, ACO has attracted an increasing number of academics, and several interesting applications are now accessible. In addition, a large body of theoretical discoveries is emerging, providing researchers and practitioners with useful advice for future ACO implementations.

**Fig. 2:**
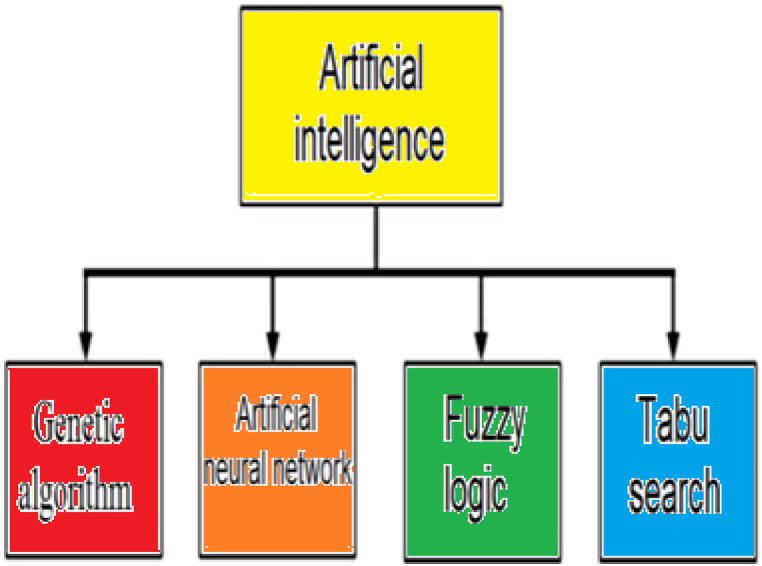
Types of artificial intelligence methods used in sizing [18].

Fig. 2 shows the four subgroups of genetic algorithm, artificial neural network, fuzzy logic and tabu search from the artificial intelligence group utilized in sizing the energy systems.

## 3 Methodology

Hybrid Optimization Model for Electric Renewables software (HOMER), which is developed by the National Renewable Energy Laboratory (NREL), has been utilized in this study and the input variables used in the simulation are mentioned in the following subsections.

### 3.1 Load demand data

**Fig. 3:**
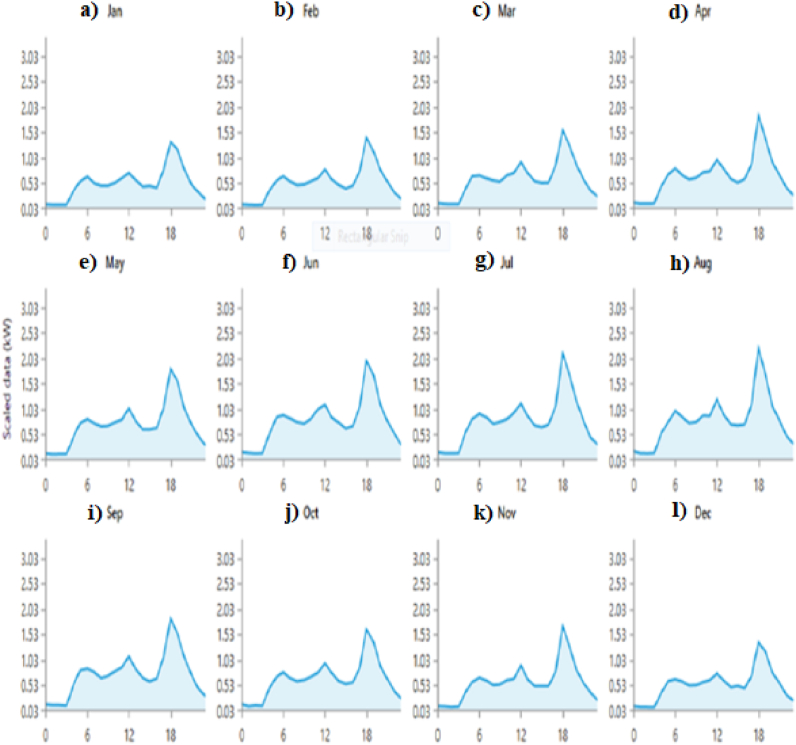
Simulation of load consumption of a house with HOMER software for a) January b) February c) March d) April e) May f) June g) July h) August i) September j) October k) November l) December.

Today, sizing using computer software is the most widely used among different sizing methods, and the reason for this is the high accuracy and convenience of these methods. The most famous computer software that has significant efficiency in the field of sizing is the software designed by the American National Renewable Energy Laboratory and provided for free to the public, HOMER and existing batteries, as well as connection or PV. In this software, by entering the data of the amount of house load and the types of sizes of non-connection of the desired section to the national power grid, the software lists the best combination and the best size of the systems in order of priority for the user. Electricity consumption in different months of the year is shown in Fig. 3.

In previous research (CS) and bird algorithm (ABC), bee algorithm (TLBO) and learning-learning algorithm (ACA) of ants have been used and the results of the optimal system size have been compared with each other by various methods. In many articles, it is assumed that the production power of the photovoltaic panel and the load profile are obtained with a prediction algorithm. Artificial neural networks have been used for load prediction. Previous researches produced scenarios to predict the load of the seasons. A coefficient for the seasonal lighting energy consumption assumed by different FCMs is increased through method 1. The forecast of solar radiation and the amount of power is/0, this coefficient is 7/in the winter season, which in the summer season, the photovoltaic panel production for the next day is based on the fuzzy method and meteorological information in this source. Another research used a robust method to predict the output power of the photovoltaic panel. Monte Carlo simulation is used to forecast wind generation and electricity demand. GAMS software to reduce scenarios in RTP, tree-based scenarios are proposed to confirm when and what technologies should work optimally to minimize the MILP stochastic programming with performance cost model and achieve the greatest reliability improvement.

**Fig. 4:**
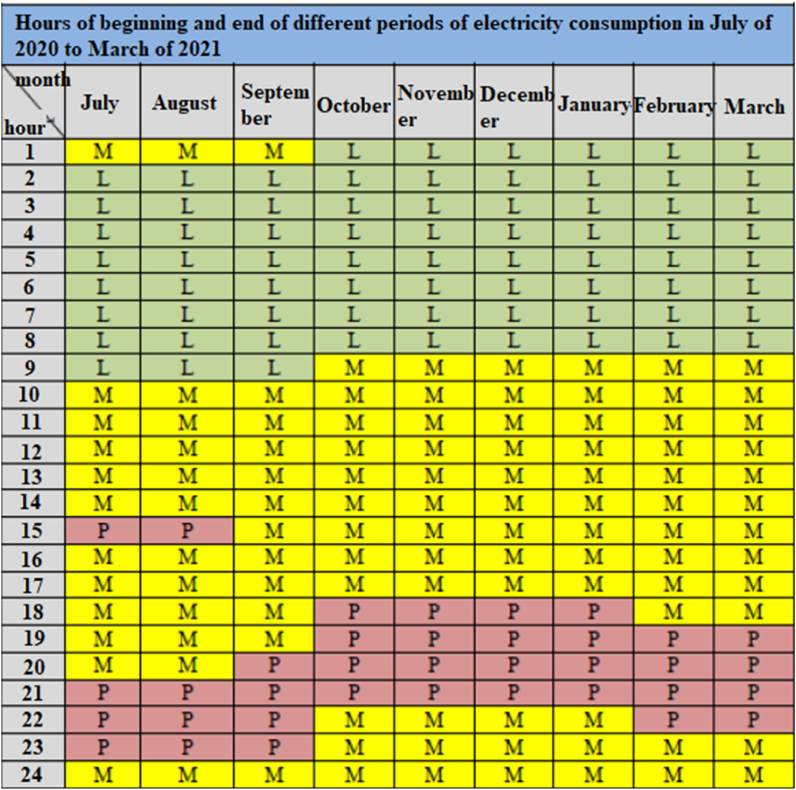
Peak load time announced by the Ministry of Energy [19] (L stands for Low load, M stands for Medium load and P stands for Peak load).

The integration of renewable energy sources into power grids has created new challenges related to its unstable nature. Therefore, different types of energy carriers (such as electricity and natural gas) are considered within the energy system to provide a certain degree of freedom of action in satisfying loads. It has been developed in an optimal management of heat and electricity for a typical residential energy system. Mathematical models of energy producers such as the combined unit of heating, cooling and electricity, household appliances such as washing machines, dryers, dishwashers, irons, pool pumps and lighting systems along with the electric car connected to the grid as an active load and thermal energy storage are presented. In this study, the objective function is to minimize all energy costs by considering the customer's preferences when using appliances. The results show the effect of integrating the load response program, the intelligent management of thermal energy storage on the reduction of energy costs in the proposed energy hub model. Energy storage is the solution to deal with the increase in the fluctuation of renewable production in the power system and also to reduce the costs of using renewable resources through peaking and shifting the load to off-peak hours. An emergency has been used. Especially when a number of different energy carriers are considered, their synergy will be able to reduce energy supply costs and increase flexibility in performance. However, these devices must be carefully selected because their installation and operation are generally expensive. In another study to investigating the effect of storage capacity and forecasting horizon on the optimal cost of energy supply of single-family houses and energy grids, it has been selected for modeling the conversion and storage of electricity, gas and heat energy carriers. Then the predictive control model is applied to determine the cost control strategy of existing conversion and storage technologies. The results obtained in this study show that in both cases of private customers and connected houses, the storage capacity and the choice of forecasting horizon are highly dependent on each other. Interest in multiple energy systems in buildings is increasing. These systems integrate different energy sources, at least one of which is renewable, to cover the required demand for electricity and heating of the building. Since the design and operation of such systems are very complex for many reasons such as the intermittent nature of renewable sources, providing a tool to help choose the best system configuration and combined energy sources is of primary importance. Fig. 4 shows the time of the day which are peak loads. Complete information on peak load, medium load and low load is also provided.

The battery system with energy 4.47 kWh was selected from the market. For this battery system the total cost is $1640 with a 4-year warranty. The battery cost is assumed $600, while other ancillary equipment cost, such as the inverter, is assumed as $1140. The battery is assumed to be capable of being fully charged and discharged, without degradation or leakage. In this project, the converter cost is estimated from the Iranian market and found to be 250 $/kW as shown in Table 1.

**Table 1:** Converter specifications.

Description	Data
Replacement cost	250 $/kW
Efficiency	96%
Lifetime	15 years

### 3.2 Solar irradiance

**Fig. 5:**
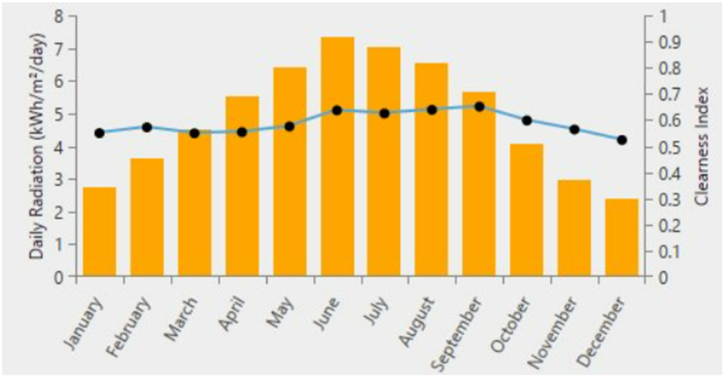
Average monthly solar radiation in Tehran, Iran.

The solar radiation in Tehran, Iran is generated by NASA's online navigator by setting the latitude as 35.7219° N and longitude as 51.3347° E. In this region as shown in Fig. 5, the mean solar radiation ranges from 2.5 kWh/m^2^ to 7.4 kWh/m^2^ each day.

### 3.3 Economic modelling

Table 2 shows the cost of photovoltaic cells in the Iranian economy.

**Table 2:** Price of solar panels system in the market [20].

PV size	Price range $	Price per kW
4	5500–7000	1700 $- 2200 $
5	7000–8000	1650 $- 1900 $
6	8000–9000	1550 $- 1750 $
10	14,000–17000	1200 $- 1500 $

The annual project cost is the difference between the saving obtained by connecting the system to the grid and the costs spent on this project as shown in equation (2).

(2) $$CoE=\frac{{Cost}_{Project}}{El\;\left(Wh\right)}$$

The cost of electricity (CoE) is estimated based on the cost of the whole project and the annual energy drawn by the load which is El. The yearly investment cost is specified as equation (3):

(3) $${Cost}_{inves,project}=\sum_{j=1}^{n-1}{Consu}_{inves,n-j}-{Cost}_{inves,extra}$$

## 4 Results and Discussion

### 4.1 Sizing

**Fig. 6:**
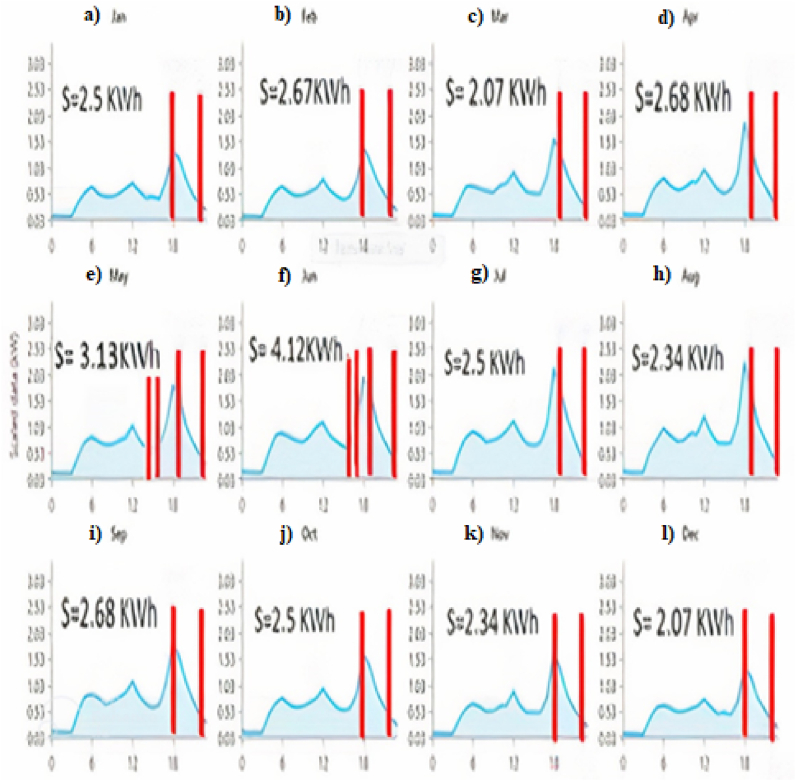
Calculation of the area under the home load curve at peak load times of the grid for a) January b) February c) March d) April e) May f) June g) July h) August i) September j) October k) November l) December.

It is very challenging to use a boost converter with dependable performance due to the increase in non-linear loads, which may result in poor power factors. As a result, traditional controllers, such as PIs, cannot control converters at all operating points. A simulation has been conducted for the power factor improvement converter circuit using parallel-connected boost converter mode, which provides close to unity power factor compensation. Based on the simulation results that were successfully carried out using Matlab Simulink software on IBC as a series of power factor improvements with fuzzy logic controllers, the power factor (PF) can be increased to close to unity power factor compensation. Based on the results, fuzzy-based controllers outperformed traditional controllers to lower overshoot and ripple voltage output. System responsiveness under varying loads was less dependent on system parameters than conventional controllers and was less susceptible to performance degradation. Therefore, the system responds dynamically even when the parameters are changed or disturbed. The use of hybrid systems in the domestic, office, commercial, etc. Sectors can provide a major part of the required energy. Supply these sectors and reduce the costs of electricity consumption, especially during peak load, so designing an optimal system to use the energy of these systems is inevitable in the developing world. This article examines various sizing methods of renewable systems. Choose the right method according to various conditions lead to the selection of the optimal system from the point of view of price and reliability. Thumb method, numerical method, method Analytical, using computer software and artificial intelligence methods are among the methods reviewed in this article. Fig. 6 shows the load curves of the home in a typical day in different months of the year.

In this paper, after finding Maximum Power Point (MPP) by P&O algorithm, a novel Fractional order Backstepping Sliding Mode Controller (FOBSMC) is designed and proposed to track the maximum power point in a PV system. The proposed method, due to the use of fractional-order calculus, was able to have a smooth output without the chattering phenomenon, on the other hand, it was able to track the maximum amount of output power in the system, which shows the high accuracy and speed of the proposed method.

### 4.2 Cost analysis

**Fig. 7:**
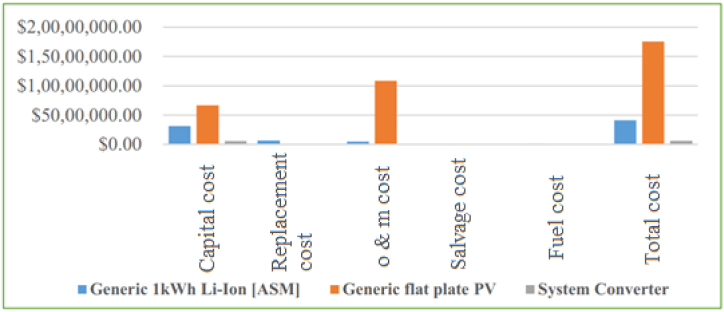
Studied combination elements NPV sharing out.

The lowest cost of electricity is noted when the capacity of the battery size is 4.5 MW with a corresponding value of 0.44 $. The lowest net present cost is achieved when the capacity of the convertor increased, it reached 15 million $ when the size becomes 4.5 MW. The COE, NPV, and investment costs are determined to be 0.44 $/kWh, 15.21 m$ and 3.39 m$ respectively. Further, in Fig. 7 the NPC for each element included in this investigation is demonstrated.

## 5 Conclusion

In this paper, the optimal placement of DGs in distribution systems was proposed. Among different kinds of DG and PV were also considered in this paper. Therefore, PEM was employed to catch the intermittence nature of such DGs. As a consequence, probabilistic load flow based on the PEM was utilized. The performance of the proposed method was evaluated using a test system. By implementing the proposed method, the site and size of each type of DG were determined regarding the problem constraints. Obtained results denoted that the system losses could be decreased by about 2.02% and the voltage of all busses located within the permitted interval. Furthermore, the proposed method showed a fast performance in convergence. The optimization based on HOMER, with economic and ecological data output shows that the least cost of electricity and net price cost are 0.44 $ per kWh and 15.0 million $ respectively, when the converter size was gradually changed, with a renewable fraction of 46.7%.

## Declarations

### Author contribution statement

Amir Mohammad Mirzaei, Iman Malek: Contributed reagents, materials, analysis tools or data; Mohammad Vahabi Khah: Conceived and designed the experiments; Contributed reagents, materials, analysis tools or data; Rahim Zahedi, Nima Rezaei: Performed the experiments; Wrote the paper; Reza Eskandarpanah, Omid Noudeh Farahani: Analyzed and interpreted the data.

### Funding statement

This research did not receive any specific grant from funding agencies in the public, commercial, or not-for-profit sectors.

### Data availability statement

Data will be made available on request.

### Declaration of interest's statement

The authors declare no competing interests.
